# Short-term stimulation with histone deacetylase inhibitor trichostatin a induces epithelial-mesenchymal transition in nasopharyngeal carcinoma cells without increasing cell invasion ability

**DOI:** 10.1186/s12885-019-5482-y

**Published:** 2019-03-22

**Authors:** Zhihua Shen, Xiaomin Liao, Zhongming Shao, Muyin Feng, Jianling Yuan, Sisi Wang, Siyuan Gan, Yanping Ha, Zhiwei He, Wei Jie

**Affiliations:** 1 Department of Pathophysiology, School of Basic Medical Sciences, Guangdong Medical University, No.2 Wenming Eastern Road, Xiashan District, Zhanjang, 524023 People’s Republic of China; 2 Department of Pathology, School of Basic Medical Sciences, Guangdong Medical University, No.2 Wenming Eastern Road, Xiashan District, Zhanjiang, 524023 People’s Republic of China

**Keywords:** Nasopharyngeal carcinoma, Histone deacetylase inhibitor, Trichostatin a, ITSA-1, Epithelial mesenchymal transition, Proliferation, Migration

## Abstract

**Background:**

Epithelial-mesenchymal transition (EMT) may be one of the reasons for the failure in some clinical trials regarding histone deacetylase inhibitors (HDACIs)-treated solid tumors. We investigated the effects of a pan-HDACI trichostatin A (TSA) on the proliferation and EMT of nasopharyngeal carcinoma (NPC) cells.

**Methods:**

Poorly-differentiated NPC cell line CNE2 and undifferentiated C666–1 were treated with various concentrations of TSA, the cell viability was assessed by CCK-8 assay, the morphology was photographed, and the mRNA level of HDACs was assessed by semiquantitative PCR. After determination the cell cycle distributions, cells were subjected to western blotting analysis of cell cycle and EMT-associated genes expression. And the changes in migration ability were assessed by transwell migration assay and scratch wound healing assay. Finally, histone deacetylases activator ITSA-1 was used to assess the reverse of TSA-induced changes in NPC cells.

**Results:**

TSA inhibited the proliferation of CNE2 and C666–1 cells in a concentration-dependent manner and arrested the cell cycle at G1 phases. TSA reduced PCNA, cyclin D1, cyclin E1, CDK2, p16 and p21 expressions and stimulated CDK6 levels. TSA stimulation for 48 h could effectively induce the EMT in CNE2 and C666–1 cells, which showed an increase of spindle-like cells and promoted expression of Vimentin and Snail1 expression in a concentration-dependent manner. Surprisingly, this short period of TSA treatment that induced EMT also impeded the migration ability of CNE2 and C666–1 cells. Interestingly, ITSA-1 rescued TSA-impeded CNE2 and C666–1 cells’ proliferation, migration and HDACs expression, also re-induced the cells to turn into epithelial cell phenotypes.

**Conclusions:**

These results indicate that short-term stimulation of TSA effectively inhibits cell proliferation and induce EMT-like changes in NPC cells but not increase its invasion ability.

## Background

Modulation of the acetylation status of histones is a crucial mechanism for the regulation of gene expression and is precisely mediated by the activity of histone acetyltransferases and histone deacetylases (HDACs) [1, 2]. HDACs are a class of enzymes that remove acetyl groups from a *ε*-N-acetyl lysine amino acid on a histone, which leads to chromatin condensation [3]. HDACs are classified into four classes based on their function and DNA sequence similarity based on sequence homology to yeast [4]: Class I (HDAC1, 2, 3, 8), Class II (HDAC4, 5, 6, 7, 9, 10) and Class IV enzymes (HDAC11) are considered classical HDACs and have a zinc-dependent active site, whereas Class III enzymes are a family of NAD^+^-dependent proteins known as sirtuins.

Histone deacetylase inhibitors (HDACIs) are chemical compounds that inhibit HDACs by blocking acetyl groups remove from the lysine residues [5]. An increasing number of HDACIs are being designed and tested for clinical use [6], and several HDACIs are being investigated in Phase I, II and III clinical trials for cancer. Early reports on HDACIs in the treatment of hematological malignancies achieved encouraging results [7, 8]. Panobinostat (LBH589), Givinostat (ITF2357) and Mocetinostat (MGCD0103) are currently in Phase II and III lymphoma or leukemia clinical trials [9–12]. In addition, several HDACIs are being used for treatment of solid tumors. For example, SB939 is entering a phase II trial for prostate cancer [13]. However, other studies have reported disappointing results for HDACIs in patients with solid tumors [14–18]. The exact mechanism underlying the cause of treatment failure of HDACIs is still unknown.

Nasopharyngeal carcinoma (NPC) is a malignant tumor arising from the mucosal epithelium of the nasopharynx. NPC is one of the most common malignant tumors in Southern China. The nonkeratinizing undifferentiated form of NPC that is tightly associated with Epstein-Barr virus infection is the most common regional histological type in Southern China [19]. Chemoradiotherapy is currently the mainstream treatment for NPC [20]. However, the use of neoadjuvant therapy for NPC is increasing. Histone modification has been considered an attractive approach for NPC adjuvant treatment [21]. Several reports have showed that HDACIs exhibit in vitro or in vivo activity against NPC [22–27], and this treatment strategy warrants further investigation. Indeed, there is one ongoing clinical trial regarding the use of HDACIs for NPC (https://clinicaltrials.gov/, NCT01341834).

Trichostatin A (TSA), one of the first generation of HDACIs, belongs to hydroxamic acids, could none selectively block the activity of Class I, II and IV HDACs, which displays the potentials in anti-cancer activity. This study aimed to determine the biological effects of TSA on NPC cells in vitro and lay a foundation for the future clinical application of HDACI in NPC adjuvant treatment.

## Methods

### Cell lines and drug treatment

The poorly differentiated human NPC cell line CNE2 (accession: SAMN07672738, NCBI BioSample) and undifferentiated C666–1 (accession: SAMN04240766, NCBI BioSample) were presented by the Cancer Institute of Southern Medical University, and we routinely test whether cells are contaminated with mycoplasma before experiments. We have not examined its STR profiles. Cells were maintained as described previously [28, 29]. Briefly, CNE2 and C666–1 cells were cultured with RMPI-1640 medium (Hyclone, Beijing, China) supplemented with 10% fetal bovine serum (FBS, Hyclone), 100 U/ml penicillin and 100 μg/ml streptomycin. Cells were incubated at 37 °C, 5% CO_2_ in a saturated humidity incubator. Medium was changed every 2 days, and cells in logarithmic growth phase were used for experiments. HDACs inhibitor TSA (#Cat.V900931, Sigma-Aldrich, Shanghai, China) was dissolved in dimethyl sulfoxide (DMSO), and its final concentrations in complete medium were 0, 50, 100, 200 and 400 ng/ml. For reversing the effects of TSA, a TSA inhibitor ITSA-1 (#Cat. HY-100508, MCE, Shanghai, China) was added in TSA-enriched medium, and ITSA-1’s final concentration was 10 μmol/l. For short-term TSA treatment, medium was changed every 48 h; cells were treated with TSA within 0–72 h then subjected to associated analysis.

### CCK-8 assay

CNE2 and C666–1 cells were seeded into 96-well plates (4000 per well). After overnight incubation, medium was replaced with medium containing TSA with or without ITSA-1 for an additional 0, 24 and 48 h. Cells were washed with 1× PBS and then 100 μl CCK-8 reagent (Beyotime Institute of Biotechnology, Nanjing, China) and RMPI-1640 medium (CCK-8 reagent: RMPI-1640 = 1:10, *v*/v) was added per well. Cells were then incubated at 37 °C for 3 h. The optical density was measured using a Multiskan MKS microplate reader (Thermo Scientific, Grand Island, NY, USA) at a wavelength of 450 nm. Each experimental treatment was repeated in 6 wells, and data represent the mean ± SEM of three independent experiments.

### RNA extraction, reverse transcription and PCR

RNA extraction, reverse transcription and quantitative PCR reactions were performed as described previously [30]. Briefly, total RNA was extracted using Trizol reagent (Invitrogen, Carlsbad, CA, USA), and 500 ng total RNA was used to generate the first strand cDNA using the Fermentas RT System (Cat. #K1622, Thermo Scientific). Sequences of primers (5′-3′, synthesized by Sangong BioTech, Shanghai, China) are listed following, *E-cadherin* (NM_004360.4), TTGCTACTGGAACAGGGACAC/CCCGTGTGTTAGTTCTGCTGT; *Vimentin* (NM_003380.4), TGCGTGAAATGGAAGAGAACT/TCAGGTTTCAGGGAGGAAAAGT; *Snail1*(NM_005985.3), CCAG AGTTTACCTTCCAGCAG/GACAGAGTCCCAGATGAGCA;*Twist1* (NM_000474), GAGCAAGATTCAGACCCTCAAG/CCATCCTCCAGACCGAGAAG; *HDAC1* (NM_004964.3), ACTGCTAAAGTATCACCAGAGGG/CACACTTGGCGTGTCCT TTG; *HDAC2* (NM_001527.4), CCAAAGGAACCAAATCAGAACAGC/TGTCAT TAGCCACTGAAACAAGAC; *HDAC3* (NM_003883.4), CTTCCTGCAGAGAGT CAGCC/GCCAGAGGCCTCAAACTTCT; *HDAC4* (NM_006037.3), ACTTGTGGGTTACCTGGCTC/TGTTGTTGCTTGATGTGCTCG; *HDAC5* (NM_001015053.1), GAGTCGGCAGATGGGATGTC/TGGGCTCCTTTGACTTCGAC; *HDAC6* (NM_ 001321225.1), CCAGAAACTTGGTGGAGCGA/TCAGATCCATCCCTTGCAGTC; *HDAC7* (NM_015401.5), CTCTCGCCGTCTCACAGTC/TACAGCACTTCGCTTGCTCT; *HDAC8* (NM_018486.3), CAGAAGGTCAGCCAAGAGGG/GACACGTCACCTGTTCCTGG; *HDAC9* (NM_178423.2),GCAACAAAACCCTAGCAGCC/CACTGCCCTTTCTCGTCCTC; *HDAC10* (NM_032019.6), TGACCCCAGCGTCCTTT ACT/TGGCTGAGTCAAATCCTGCC; *HDAC11* (NM_024827.4), CCCCGGGATGCTACACAC/ACGCTTGTCGTCCATGAAGT; *β-actin* (BC002409), TGACGTGGACATCCGCAAAG/CTGGAAGGTGGACAGCGAGG. For quantitative PCR, the reaction conditions were pre-denaturation at 95 °C for 5 min, followed by 45 cycles of 95 °C for 10 s and 60 °C for 30 s. The relative abundance of target genes were determined from the C_T_ values and plotted as the fold change compared with the control groups (2^-∆∆Ct^). For semiquantitative PCR, the reaction conditions were pre-denaturation at 95 °C for 4 min, followed by 35 cycles of 95 °C for 15 s, 60 °C 30 s and 72 °C for 45 s. For all PCR analysis, the levels of *β*-actin mRNA served as an internal control.

### Western blot

Cells were collected and lysed with protease and phosphatase inhibitor-containing RIPA buffer (#P0013C, Beyotime Institute of Biotechnology). Total proteins (30–50 µg) were subjected to SDS-PAGE and then proteins were transferred to the polyvinylidene difluoride membranes. After two washes with TBST, the membranes were incubated with 5% skimmed milk in TBST at 37 °C for 2 h. The membranes were then incubated with the following primary antibodies: PCNA (1:1000, #2586, Cell Signaling, Danvers, MA, USA), E-cadherin (1:1000, #14472, Cell Signaling), Vimentin (1:1000, #3390, Cell Signaling), Snail1 (1:500, #13099–1-AP, ProteinTech, Wuhan, China), Twist1 (1:500, #25465–1-AP, ProteinTech), cyclin D1 (1:500, #60186–1-Ig, ProteinTech), cyclin B1 (1:500, #55004–1-AP, ProteinTech), cyclin E1 (1:500, #11554–1-AP, ProteinTech), CDK2 (1:500, #10122–1-AP, ProteinTech), CDK4 (1:500, #11026–1-AP, ProteinTech), CDK6 (1:500, #14052–1-AP, ProteinTech), p16 (1:500, #10883–1-AP, ProteinTech), p21 (1:500, #10355–1-AP, ProteinTech) and β-actin (1:2000, #60008–1-Ig, ProteinTech). After two washes with TBST, the membranes were incubated with horseradish peroxidase (HRP)-conjugated IgGs (1:3000, Santa Cruz, Dallas, TX, USA) overnight at 4 °C. Bands were visualized by using enhanced chemiluminescence reagents (Thermo Fisher, Rockford, IL, USA) and analyzed with a gel analysis system (Tanon, Shanghai, China).

### Determination of cell cycle distribution

CNE2 and C666–1 cells were seeded in 6-well plates and treated with TSA (0 and 200 ng/ml) for 48 h. At least 1 × 10^6^ cells were harvested and washed with cold PBS; cells were subsequently stained with propidium iodide and subjected to flow cytometric analysis (BD FACSCanto II, Franklin Lakes, NJ, USA). Each experimental group was repeated in three wells.

### Transwell migration assay

Transwell migration assay was performed as previously described using 8-μm pore size polycarbonate membranes chambers (Corning, Tewksbury, MA, USA) [29, 30]. Briefly, 48 h following treatment with TSA (0 and 200 ng/ml), CNE2 and C666–1 cells were harvested in RMPI-1640 supplemented with 0.5% FBS. Cells (2 × 10^4^ cells in total 200 μl) were seeded in the upper chamber; the lower chamber contained 500 μl RMPI-1640 medium supplemented with 10% FBS. Migration was allowed to proceed for 24 h at 37 °C. The insert chambers were fixed for 15 min with 4% paraformaldehyde and stained with 0.1% crystal violet. Membranes were washed with PBS. Cells on the upper surface of the membrane were removed with a cotton swab. The images were photographed with a microscope (Leica, Wetzlar, Germany) at 20× objective fields, and the number of migrated cells on the lower surfaces of the membranes was counted. Each experimental group was repeated in three chambers, and cell numbers from a total of nine representative fields were obtained.

### Scratch wound healing assay

CNE2 and C666–1 cells were seeded into 6-well plates and cultured until they reached ~ 100% confluence as a monolayer. The monolayer was scratched with a 100 μl pipette tip across the center of the well, and a total of three wound gaps were made in parallel for each well. The wells were washed with PBS to remove the detached cells and then 2 ml complete medium supplemented with TSA (0 or 200 ng/ml) or TSA + ITSA-1 (TSA, 200 ng/ml; ITSA-1, 10 μmol/l) was added. Images were photographed at 0, 24 and 48 h later. The scratch area was measured using the Image J software (https://imagej.nih.gov/ij/index.html). All experiments were repeated in triplicate.

### Statistical analyses

Data were expressed as the mean ± SEM. Statistical analyses were performed using PRISM Software 7.0 (GraphPad Software, CA, USA). Student *t* test and ANOVA was performed accordingly. For all analyses a two-sided *P*-value of < 0.05 was considered statistically significant.

## Results

### TSA significantly inhibits NPC cells proliferation

**Fig 1:**
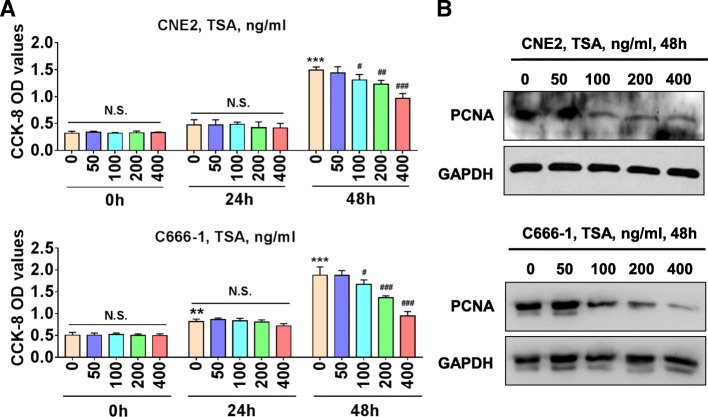
TSA inhibits NPC cells proliferation. **a** CNE2 and C666–1 cells plated in 96-well plate were treated with the indicated various concentrations of TSA for 0 h, 24 h and 48 h, and CCK-8 assay was performed. N.S., no significance; **P < 0.01 vs. 0 h; ***P < 0.001 vs. 0 h; ^#^*P* < 0.05 vs. TSA 0 ng/ml; ^##^*P* < 0.01 vs. TSA 0 ng/ml; ^###^*P* < 0.001 vs. TSA 0 ng/ml. **b** Western blot of PCNA protein expression in CNE2 and C666–1 cells treated with various concentrations of TSA for 48 h

We first performed CCK-8 assay to test the effects of TSA on CNE2 and C666–1 cells’ viability. Compared with the 0 ng/ml group, 24 h TSA treatment of CNE2 and C666–1 cells showed no any inhibitory effects on cell’s viability, whereas 48 h TSA treatment led to significantly attenuated cell viability at a range of TSA concentrations from 100 ng/ml to 400 ng/ml (Fig. 1a). We also examined PCNA protein expression as a marker for cell proliferation using western blot. As shown in Fig. 1b TSA (100–400 ng/ml, 48 h) also reduced PCNA protein expression in CNE2 and C666–1 cells. Together these results indicate that TSA stimulation can inhibit the proliferation of NPC cells.

### TSA stimulation blocks NPC cell cycle progression through disturbing cell cycle-associated protein expression

**Fig 2:**
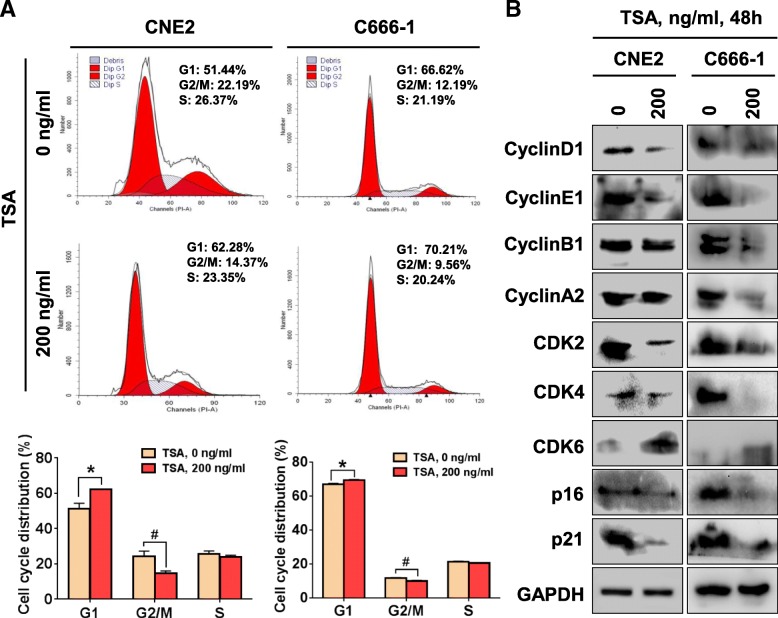
Effects of TSA on the cell cycle distribution of NPC cells**. a** Flow cytometric analysis was performed to analyze the cell cycle distribution of CEN2 and C666–1 cells treated with 0 and 200 ng/ml TSA for 48 h. *P < 0.05; ^#^*P* < 0.05. **b** Western blot analysis was performed for the indicated proteins in CNE2 and C666–1 cells treated with 0 and 200 ng/ml TSA for 48 h

We next examined the effects of TSA on cell cycle of CNE2 and C666–1 cells. We found that compared with the control group, both CNE2 and C666–1 cells treated with 200 ng/ml TSA for 48 h showed a significantly increased percentage of cells in G1 and decrease in G2/M phases but not the S phase (Fig. 2a). The cell cycle is precisely regulated by the activities of various cyclins, cyclin dependent kinases (CDKs) and cyclin dependent kinase inhibitors (CDKIs), and we thus further performed western blotting to analyze the changes in proteins involved in cell cycle regulation. As shown in Fig. 2b, TSA (200 ng/ml, 48 h) treatment decreased cyclinD1, cyclinE1, CDK2, CDK4, p16 and p21 expression and increased CDK6 expression in both CNE2 and C666–1 cells; however, TSA showed no effects on cyclinB1 and cyclinA2 expression in CNE2 cells but displayed inhibitory effects in C666–1 cells. These results illustrated the complexity of the effects of TSA on NPC cell cycle-related genes expression.

### TSA stimulation induces mesenchyme-like phenotype in NPC cells

**Fig 3:**
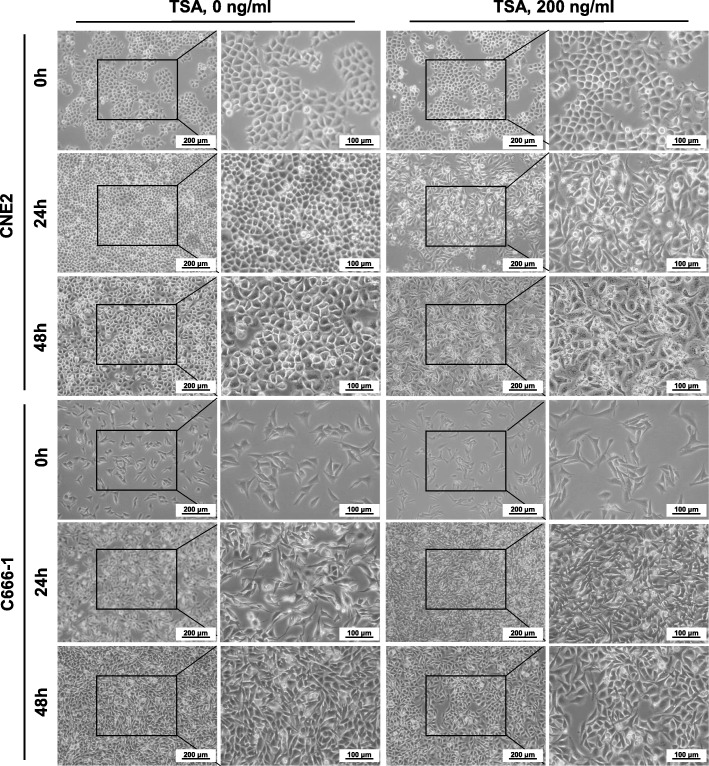
Morphology of NPC cells treated with TSA. CNE2 and C666–1 cells were treated with 0 and 200 ng/ml TSA for 0, 24 and 48 h, and then cells were photographed under inverted microscope. Scale bars = 200 μm or 100 μm

The untreated CNE2 cells showed a polygonal morphology and the confluent monolayers were arranged in a stone paving way (Fig. 3). However, after treatment with 200 ng/ml TSA for 24 h, almost all cells were spindle-shaped. In response to a 48 h of 200 ng/ml TSA, the confluence of cells was little decreased, with part of the cells showing cytoplasmic vacuoles and atypical spindle-like cell morphology, including pseudopodia-like structures. These changes in cell morphology suggested that TSA stimulates CNE2 cells to induce EMT changes. As to the undifferentiated C666–1 cells, the changes in morphology seemed not to be such obvious like to CNE2 cells, for it has already displayed short spindle-like cell morphology before TSA treatment. Even this, we could observe TSA-treated C666–1 cells have turned into much thinner and longer shapes than before (Fig. 3).

### TSA alters EMT-related gene expression in NPC cells

**Fig 4:**
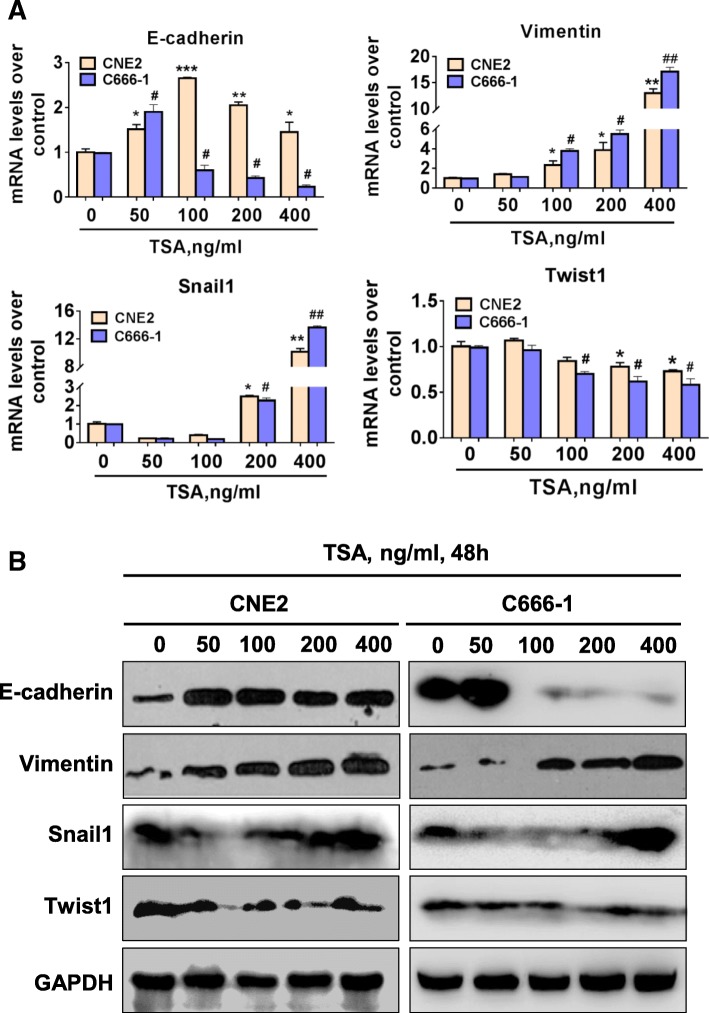
TSA induces EMT-associated marker expression in NPC cells in a concentration-dependent manner**.** CNE2 and C666–1 cells were treated with various concentrations of TSA for short period within 48 h, and then cells were harvested and subjected to real-time PCR **a** and western blot **b** analysis of EMT-associated E-cadherin, Vimentin, Snail1 and Twist1 gene and protein expressions. *P < 0.05 vs. TSA 0 ng/ml; **P < 0.01 vs. TSA 0 ng/ml; ***P < 0.001 vs. TSA 0 ng/ml; ^#^*P* < 0.05 vs. TSA 0 ng/ml; ^##^*P* < 0.01 vs. TSA 0 ng/ml

We next examined the mRNA and protein expressions of various EMT-related factors, including E-cadherin, Vimentin and Snail1 and Twist1, in CNE2 and C666–1 cells by qPCR and western blotting in response to TSA treatment. The results showed that TSA stimulation for 48 h induced the expression of mesenchymal marker gene *Vimentin*; moreover, we found that TSA upregulated *Snail1* but inhibited *Twist1* expression by a concentration-dependent manner. Regarding the expression of epithelial marker *E-cadherin*, we observed a trend of increasing first then following decrease later (Fig. 4).

### Short terms of TSA stimulation suppresses the migration of NPC cells

**Fig 5:**
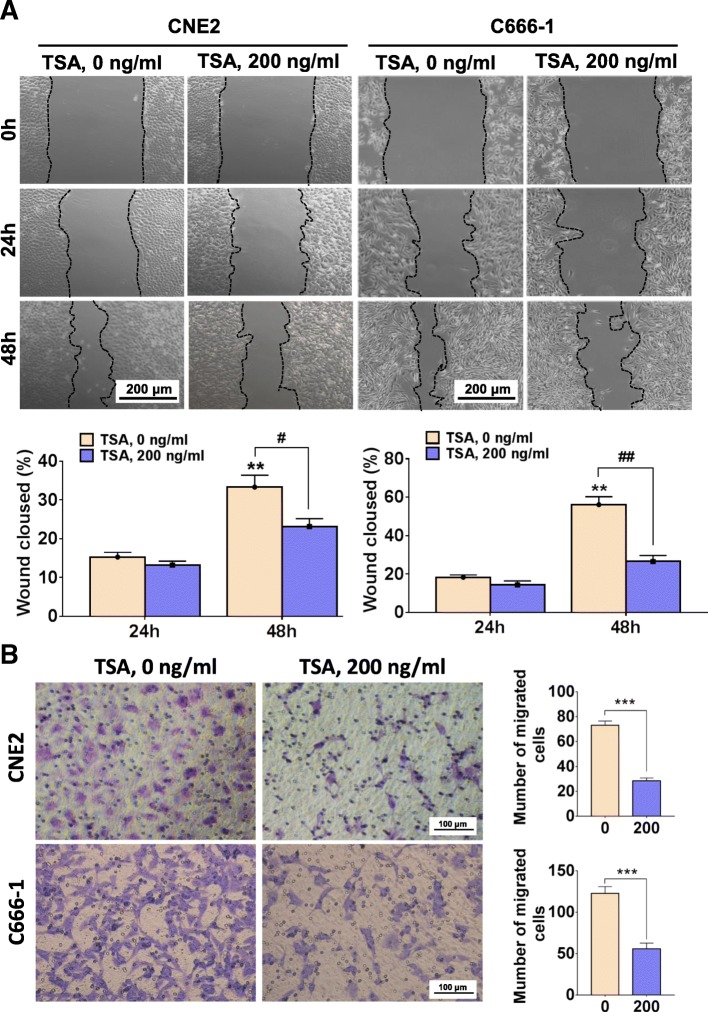
TSA attenuates NPC cells motility within short periods of treatment. CNE2 and C666–1 cell were treated with 0 and 200 ng/ml TSA for 48 h and scratch wound healing assay **a** and transwell migration assay **b** were performed. In **a** **P < 0.01 vs. 24 h, ^#^*P* < 0.05; ^##^*P* < 0.01; in **b** ***P < 0.001

In general, the appearance of EMT phenotype in tumor cells implies an increase in cell migration capacity. We used both transwell chamber migration assay and scratch injury repair assay to examine the migration ability of CNE2 and C666–1 cells treated with TSA. In contrast to our expectation, although TSA induced EMT-like changes in the morphology of CNE2 and C666–1 cells, its migration abilities were both reduced in response to 200 ng/ml TSA for 48 h (Fig. 5). We observed a significant decrease in the number of cells on the upper surface of the chamber membrane and the weakened repair of scratched lesion areas compared with the control groups (Fig. 5).

### ITSA-1 reverses TSA-induced morphological and biological function changes in NPC cells

**Fig. 6:**
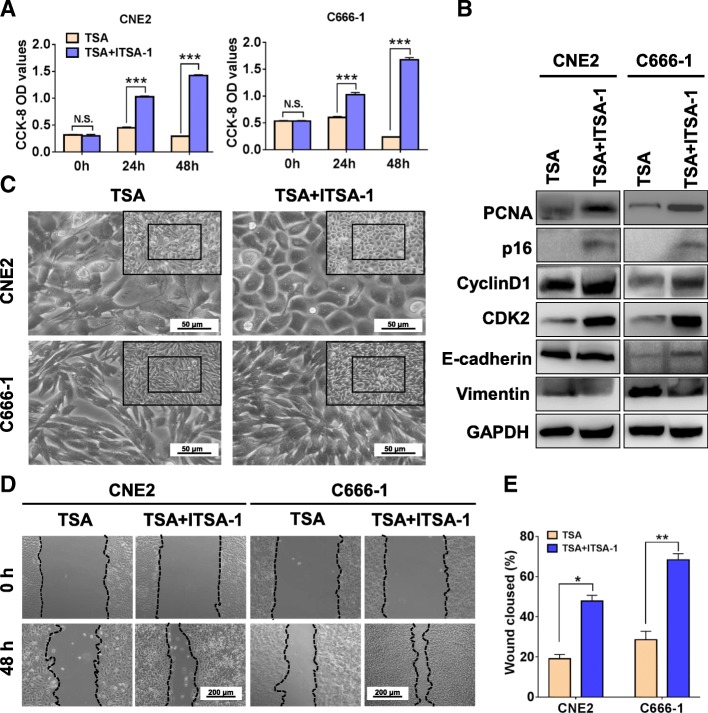
ITSA-1 reverses TSA-impacted changes in NPC cells**. a** CCK-8 assay was used to detect the cell viability of TSA (200 ng/ml) and TSA + ITSA-1(10 μmol/l) - treated CNE2 and C666–1 cells. N.S., no significance; ***P < 0.001. **b** Western blot was used to examine the protein levels of cell proliferation (PCNA), cell cycle (cyclinD1, CDK2 and p16) and EMT-associated genes (E-cadherin and Vimentin). **c** Morphology of TSA and ITSA-impacted NPC cells. Scale bars = 50 μm. **d** & **e** Scratch wound healing assay was performed to test the migration ability of CNE2 and C666–1 cell treated with TSA or TSA + ITSA-1 for 48 h. Scale bars = 200 μm. *P < 0.05; **P < 0.01

To further confirm that the effects of TSA on NPC cells, we adopted ITSA-1, an suppressor for TSA [31], to recover the morphological and biological changes of TSA-impacted on NPC cells. As shown in Fig. 6, ITSA-1 obviously reversed TSA-attenuated cell proliferation (Fig. 6a) and induced mesenchymal morphological changes (Fig. 6c). We also found that ITSA-1 significantly altered the molecular levels in cell proliferation (PCNA), cell cycle (p16, cyclinD1 and CDK2) and EMT-associated genes (E-cadherin and Vimentin) post TSA treatment (Fig. 6b). And these changes were well matched with its enhanced migration ability that shown in Fig. 6d. Together these results, ITSA-1 effectively reversed the morphological and biological changes of TSA-impacted on NPC cells.

### HDACs expression and role in TSA and ITSA-1-treated NPC cells

**Fig 7:**
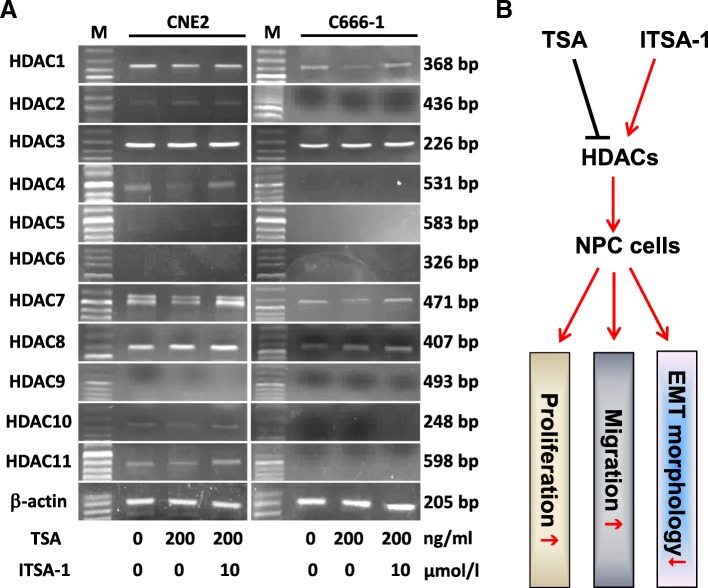
Expression and functional role of HDACs in TSA and ITSA-1-treated NPC cells. **a** Semiquantitative PCR was used to detect the mRNA level of HDAC1, 2, 3, 4, 5, 6, 7, 8, 9, 10 and 11 in TSA and TSA + ITSA-1 treated CNE2 and C666–1 cells. M, DNA marker. **b** Schematic diagram of the functional mechanisms of TSA and ITSA-1-impacted HDACs on NPC cells

Because of TSA was an inhibitor for panels of HDACs including class I (HDAC1, 2, 3, 8), class II (HDAC4, 5, 6, 7, 9, 10) and class IV (HDAC11). We thus examined the changes in mRNA levels of HDACs post TSA-treated NPC cells using semiquantitative PCR. As shown in Fig. 7a, the expressions of basic levels of HDACs were various in CNE2 and C666–1 cells. TSA attenuated most of HDACs including HDAC1, HDAC2, HDAC4, HDAC7, HDAC10 and HDAC11 in CNE2 cells and HDAC1 and HDAC7 in C666–1 cells to a certain extent, whilst ITSA-1 enhanced their levels. We observed even HDAC3 and HDAC8 highly expressed in both CNE2 and C666–1 cells, TSA showed no significantly effects on its expressions. Considering the crucial roles of HDACs in cell biology, we generalized the potential mechanisms of TSA and ITSA-1 on NPC cells’ proliferation, migration and EMT morphology, as elucidated in Fig. 7b.

## Discussion

As one of the first generation HDACIs, TSA has been reported to exhibit anti-cancer activity in many human solid tumors [32–34]. To examine the potential activity of TSA in human NPC, we used TSA to treat the poorly differentiated human NPC cell line CNE2 and undifferentiated C666–1 cells and found that TSA effectively attenuated both CNE2 and C666–1 cell proliferation, as evidenced by the suppressed cell viability and PCNA expression. Our results indicate that TSA exhibits anti-cancer properties in human NPC cells.

Because the altered cell proliferation capacity may result from changes in the cell cycle, we next analyzed the cell cycle distribution of CNE2 and C666–1 cells in response to TSA. CNE2 and C666–1 cells treated with TSA displayed an increased percentage of cells in G1 phase and decreased percentage of cells in G2/M phase. We speculated that TSA-mediated inhibition of CNE2 and C666–1 cell proliferation may results from its ability to arrest cells in G1 phase. We performed western blotting to analyze the expressions of cyclins, CDKs and CDKIs and found that TSA treatment of CNE2 and C666–1 cells led to a decrease in cyclinD1, cyclinE1, CDK2, CDK4, p16 and p21 expression and increased CDK6 expression in both CNE2 and C666–1 cells; however, with no changes in cyclinB1 and cyclinA2 expression in CNE2 cells but a inhibitory in C666–1 cells. As a dogma, the cyclinD-CDK4/6 complex controls G1 phase progression, the cyclinE-CDK2 complex controls S phase progression, the cyclinA-CDK2 complex controls G2 phase progression and the cyclinB-CDK1 complex mediates M phase progression. In our results, although we detected a decrease of cyclinD1 and CDK4 levels in response to TSA, we also observed decreased p16 and p21. We guess that inhibition of p16/p21 and increase of CDK6 may not sufficiently help cells overcome the deficiencies of cyclinD1-CDK4/6 complex in TSA-treated NPC cells. This may be the reason that TSA-treated CNE2 and C666–1 cells were arrested at G1 phase. Previous reports have shown that TSA impacts cell cycle distribution. Saini et al. reported that TSA synchronized bovine embryos at G0/G1 stage [35], and Wang et al. showed that TSA arrested bladder cancer cells at G2/M and G1 phase [36]. In addition, Alao et al. found that TSA arrested breast cancer cells at G1/S or G2/M phase depending on the estrogen receptor alpha level [37]. Our present study provided further evidence that TSA-altered cell cycle progression is cell type-dependent. Taken together, our findings indicate that TSA treatment of CNE2 and C666–1 cells alters cell cycle-associated protein expression.

To our surprise, TSA treatment reduced the number of CNE2 and C666–1 cells and the remaining cells underwent EMT-like morphological changes, which were most obviously observed in epithelial CNE2 cells. Previous studies indicated that EMT endows tumor cells much stronger potentials in local invasion and distant metastasis activities [38]. These results inspired us to hypothesize whether the appearance of EMT-like cells is one of the mechanisms for HDACI treatment failure in solid tumors. We evaluated EMT markers in TSA-treated cells, and surprisingly, a short period of TSA treatment increased the expressions of the mesenchymal marker Vimentin both at mRNA and protein levels in CNE2 and C666–1 cells. In response to short TSA treatment periods, the epithelial marker E-cadherin expression showed a trend of decrease followed by an increase in these cells. Both Snail1 and Twist1 are key transcription factors that meditate EMT in cancer [39], and Snail1 is a key suppressor for E-cadherin [40]. We found that the trends of E-cadherin and Snail1 expression in TSA-treated NPC cells were just opposite. Thus, TSA-induced changes in Snail1 may be the reason for E-cadherin shift in NPC cells. Twist1 was inhibited in both CNE2 and C666–1 cells, and the reason is unknown. Previous studies demonstrated that cancer cells exhibit type 3 EMT, which is different to type 1 EMT in embryonic development and type 2 EMT in inflammation and fibrosis; type 3 EMT endows cancer cells certain epithelial cell traits while obtaining a mesenchymal phenotype [41]. Our current results supported the characteristics of type 3 EMT in TSA-treated NPC cells, especial the epithelial-like CNE2 cells.

Local recurrence of distant metastasis is the main cause of tumor treatment failure, and EMT may account for the key mechanism for failure in HDACI-treated clinical patients with solid tumors [14–18]. There are two distinct perspectives on the role of TSA-induced EMT in tumor cells. Some reports showed that TSA contributes to EMT in cancers [42–44]; however other reports proposed the opposite conclusions [45–47]. This inconsistency suggests a diversity of TSA-induced effects on EMT on various types of tumors. In our study, we also observed an EMT in NPC cells responded to TSA treatment. EMT is considered to play a positive role in promoting cancer cell invasion and migration. Interestingly, despite the occurrence of EMT in NPC cells treated with TSA in this study, we observed that TSA treatment led to attenuated cell motility of both CNE2 and C666–1 cells. These results regarding TSA inhibition of NPC motility are in conflict with the classical roles of EMT in cancer. The exact mechanism remains elusive. Previous reports indicated that TSA could suppress gastric cancer, breast cancer and cholangiocarcinoma cell migration [42, 43], and our results are consistent with these previous reports. We found that the expression of E-cadherin was first increased and then decreased after TSA treatment of CNE2 cells in a short period of time (within 48 h). We wonder whether long-term TSA treatment of NPC cells will eventually lead to a decline in tumor invasion ability, and this question is worthy of further investigation.

To further confirm the biological effects of TSA on NPC cells, we used another inhibitor named ITSA-1 to test again. ITSA-1 is a small molecule, and which has been successfully tested in rescue histone acetylation and transcriptional activation [31, 48]. As mentioned above, TSA inhibited NPC cells’ viability, distributed cell cycle and EMT-associated genes expression, and in turn lead to morphological changes and impaired cell migration, which could be significantly reversed by ITSA-1. Mechanically, various type of HDACs including HDAC1, 2, 3, 4, 5, 6, 7, 8, 9, 10, 11 were examined in TSA and ITSA-1-treated NPC cells, and we found that due to difference in the basic genetic backgrounds, poorly differentiated CNE2 (Epstein-Barr virus negative due to massive passage) and undifferentiated C666–1 (Epstein-Barr virus positive) displayed differentially HDACs expression properties. However, TSA could suppress most of the HDACs in NPC cells to a certain degree, which was well matched with its role for a pan-HDACIs; on the opposite, ITSA-1 effective rescued the TSA-attenuated HDACs’ expression. Thus, we speculated that TSA and ITSA-1 may differentially impact the expression in HDACs, which in turn results in changes in cell proliferation, migration and even the EMT morphology. Collectively, this is the first reports showed that administration of ITSA-1 could reverse TSA-impacted morphological and functional changes in NPC cells, which provides a new evidence for the possible application of anti-HDACs in NPC biology.

TSA is a potent and specific inhibitor of HDACs, which showed a dose-dependently decreases HDAC activity [49]. By interacting with the catalytic site of HDACs, TSA thereby blocks substrate access. An early report showed that 0.5–1.0 mg/kg TSA was safe in mouse embryogenesis without apparent toxicity [50]. The IC50 value of 124 nM was reported for treatment of human breast carcinoma cells in vitro, and what’s more, TSA did not cause any measurable toxicity in doses of 5.0 mg/kg in rat mammary carcinoma models [51]. Generally, in vivo administration of TSA is safe. For better understand of pharmacological and toxicological effects of TSA and its hydroxamate based structural analogues, please refer to literature [52]. In our current in vitro study, the effective concentration of TSA showed anti-proliferation was 100 ng/ml, which was equivalent to 330 nM. Thus, the effective concentration of TSA is cell type specific.

Several studies have shown that the combination of HDACIs and other reagents would be greatly beneficial for solid tumor treatment. For examples, the combination of TSA and a natural flavonolignan Silibinin could synergistically modulate EMT in human non-small cell lung cancer cells [53]. The combination of low-dose cisplatin with TSA showed greater efficacy than single drug treatment and significantly suppressed cell viability, migration, and spheroid formation and growth in ovarian cancer cells [54]. Thus, epigenetic treatment based on the application of HDACIs combined with other targeted therapies may be a new strategy for cancer therapy in the future. Regarding NPC, the combination of a proteasome inhibitor, bortezomib, and class I HDAC inhibitors potently induced killing of NPC cells both in vitro and in vivo [25]. In addition, synergistic cytotoxic effects of Abexinostat combined with cisplatin or irradiation were reported in vitro for NPC xenografts [24]. Even these preclinical studies indicated that HDACIs are effective for NPC therapy. Based on our results suggesting that HDACIs may induce EMT, HDACI treatment of NPC still needs careful consideration.

## Conclusions

In summary, our present study confirmed that a pan-HDACI TSA can significantly inhibit the proliferation of NPC cells, but at the same time TSA also induces EMT-like morphological changes. Although a short period of TSA treatment did not increase the migration ability of the surviving cells, considering that clinical administration of this drug is not a single dose but a scheduled long term, a carefully designed study of the long-term effects of TSA is needed before the application of HDACIs in clinical trials for NPC patients.

## Abbreviations

CDKIs: Cyclin dependent kinase inhibitors
CDKs: Cyclin dependent kinases
DMSO: Dimethyl sulfoxide
EMT: Epithelial-mesenchymal transition
FBS: Fetal bovine serum
HDACIs: Histone deacetylase inhibitors
HDACs: Histone deacetylases
NPC: Nasopharyngeal carcinoma
TSA: Trichostatin A
